# Development and validation of a nomogram for predicting postoperative hypocalcemia in patients undergoing surgery for differentiated thyroid cancer

**DOI:** 10.3389/fendo.2025.1628453

**Published:** 2025-10-06

**Authors:** Liangfa Zhou, Yanhua Tu, Shuanglai Qin, Qiu Zhang, Hong Ba, Ping Wang

**Affiliations:** ^1^ Department of Oncology, Wuhan No.1 Hospital (Wuhan Hospital of Traditional Chinese and Western Medicine), Wuhan, Hubei, China; ^2^ Department of Traditional Chinese Medicine, Renmin Hospital, Hubei University of Medicine, Shiyan, Hubei, China; ^3^ Department of Otorhinolaryngology, Wuhan No.1 Hospital (Wuhan Hospital of Traditional Chinese and Western Medicine), Wuhan, Hubei, China; ^4^ Department of Pediatrics, Gutian Street Community Health Service Center, Wuhan, Hubei, China; ^5^ Department of Endocrinology, Affiliated Hospital of Hubei University of Chinese Medicine (Hubei Provincial Hospital of Traditional Chinese Medicine), Wuhan, Hubei, China

**Keywords:** hypocalcemia, thyroid neoplasms, risk factors, nomograms, postoperative complications

## Abstract

**Objective:**

This study aimed to identify risk factors for hypocalcemia following differentiated thyroid cancer (DTC) surgery and develop and validate a nomogram model to predict its occurrence.

**Methods:**

A retrospective cohort study included 315 DTC patients who underwent surgery between January 2023 and January 2025. Clinical data encompassing demographics, surgical parameters (e.g., central lymph node dissection, capsular invasion, operation time), comorbidities (e.g., diabetes), and preoperative biomarkers (albumin, Lp-PLA2, Nesfatin-1) were analyzed. Variables were screened using univariate analysis, followed by multivariate logistic regression with a backward stepwise selection procedure to identify independent predictors. A nomogram was constructed using RStudio (version 3.4), and its performance was assessed for discrimination and calibration. Internal validation was performed using a bootstrapping technique.

**Results:**

Postoperative hypocalcemia occurred in 32.06% of patients (101/315). Multivariate analysis identified diabetes (OR=2.1, 95%CI: 1.4–3.3), central lymph node dissection (OR=3.4, 95%CI: 2.1–5.6), capsular invasion (OR=2.8, 95%CI: 1.7–4.7), prolonged operation time (OR=1.9, 95%CI: 1.3–3.0), elevated preoperative albumin (OR=1.7, 95%CI: 1.2–2.5), and Lp-PLA2 (OR=2.3, 95%CI: 1.5–3.7) as significant risk factors. Serum Nesfatin-1 emerged as a protective factor (OR=0.6, 95%CI: 0.4–0.9). The nomogram demonstrated excellent discrimination, with an area under the curve (AUC) of 0.850 (95%CI: 0.825–0.930), which remained robust after bootstrap validation (bias-corrected AUC=0.845). The model showed good calibration, confirmed by a calibration plot and the Hosmer-Lemeshow test (P=0.30). Decision curve analysis indicated a strong clinical utility.

**Conclusion:**

This study developed and internally validated a nomogram integrating clinical, surgical, and biochemical predictors to quantify individualized risk of hypocalcemia after DTC surgery. The model shows robust predictive accuracy, offering clinicians a practical tool for preoperative risk stratification and targeted interventions. However, external validation in multicenter cohorts is necessary to enhance generalizability.

## 1 Introduction

Hypocalcemia remains a prevalent complication following differentiated thyroid cancer (DTC) surgery, with reported incidence rates ranging from 5% to 35% for transient hypocalcemia, and 0.5–4.4% progressing to permanent hypoparathyroidism (1). While advancements in surgical techniques have reduced overall postoperative morbidity, hypocalcemia persists as a clinically significant adverse event associated with prolonged hospitalization and increased healthcare expenditure (2). The etiology of postsurgical hypocalcemia primarily involves mechanical or thermal injury to parathyroid glands during dissection, compounded by ischemic insults from compromised vascular supply (3). Extensive surgical procedures, particularly those necessitating central lymph node dissection or en bloc tumor resection, further elevate this risk due to anatomical proximity between thyroid tissue and parathyroid glands (4).

Existing studies have identified multiple risk factors, including surgical extent, preoperative calcium levels, and comorbidities such as diabetes mellitus (5, 6). However, current predictive approaches predominantly rely on isolated clinical parameters, lacking integrative models to quantify cumulative risk. Nomograms, validated graphical tools for multivariate risk stratification, have demonstrated utility in predicting oncological outcomes but remain underutilized in thyroid surgery complications (7). This methodological gap impedes preoperative identification of high-risk patients who may benefit from prophylactic interventions.

This study aimed to address these limitations by systematically analyzing perioperative variables and constructing a clinically applicable Nomogram model. Through multidimensional assessment of biochemical markers and surgical parameters, we sought to enhance predictive accuracy for post-thyroidectomy hypocalcemia, thereby informing targeted perioperative management strategies.

## 2 Materials and methods

### 2.1 Study population

A total of 315 patients with differentiated thyroid cancer (DTC) admitted to our hospital from January 2023 to January 2025 were enrolled. An *a priori* sample size calculation was performed using G*Power software (version 3.1). Assuming a power of 0.80, an alpha of 0.05, and an anticipated area under the curve (AUC) of 0.75 for the new model versus 0.50 for the null model, a minimum of 98 patients (with approximately 32 events, based on a 32% incidence rate) were required. To accommodate a multivariate model with up to 7–8 potential predictors, we aimed for a larger cohort. The final enrolled sample of 315 patients, with 101 events (hypocalcemia cases) and 7 variables in the final model, yielded an events-per-variable (EPV) ratio of 14.4, which is above the commonly recommended threshold of 10, ensuring model stability and reducing the risk of overfitting. Inclusion criteria were as follows: ① pathologically confirmed DTC; ② underwent thyroid cancer surgery without other serious postoperative complications; ③ aged ≥18 years, regardless of gender; ④ signed informed consent and actively cooperated with the study and related tests. Exclusion criteria included: ① coexisting other thyroid diseases; ② previous abnormal blood calcium levels; ③ mental or neurological disorders; ④ recent use of drugs affecting parathyroid hormone or blood calcium levels.

### 2.2 Data Collection

Clinical data were collected through the hospital medical record system, including body mass index (BMI), comorbidities, tumor diameter, lesion scope, age, lateral cervical lymph node dissection, gender, central lymph node dissection, surgical approach, marital status, operation time, capsular invasion, and preoperative serum levels of albumin (ALB), Nesfatin-1, and lipoprotein-associated phospholipase A2 (Lp-PLA2). Initially, 332 patient records were screened for eligibility. Patients with missing data for any of the key analytical variables (n=17) were excluded from the final analysis. The final study cohort thus consisted of 315 patients with complete datasets. According to previous studies, postoperative hypocalcemia was defined as a minimum serum total calcium level <2.10 mmol/L within 3 days after surgery, and severe hypocalcemia was defined as a minimum level <1.87 mmol/L (8). Patients were divided into the occurrence group (hypocalcemia + severe hypocalcemia) and non-occurrence group based on blood calcium levels for further analysis of influencing factors. A flowchart of the study design, patient selection, and analysis process is shown in **Supplementary Figure 1**.

### 2.3 Quality control

Data entry was verified for missing items, errors, or logical inconsistencies. All data were double-entered by two independent researchers, and discrepancies were resolved by reviewing medical records to ensure data integrity and accuracy.

### 2.4 Statistical analysis

SPSS 26.0 software was used for data analysis. The normality of continuous data distribution was assessed using the Shapiro-Wilk test. Normally distributed continuous data were expressed as “mean ± standard deviation” and compared between groups using the independent samples t-test. Non-normally distributed data would have been analyzed using the Mann-Whitney U test, although all continuous variables in this study followed a normal distribution. Categorical data were presented as frequencies and percentages and analyzed using the χ² test. Fisher’s exact test was applied when the expected count in any cell was less than 5. Multivariate logistic regression models were applied to identify independent risk factors for postoperative hypocalcemia in DTC patients. All variables with a P-value < 0.10 in the univariate analysis were initially included in the multivariate model. A backward stepwise selection procedure based on the Akaike Information Criterion (AIC) was then used to select the most parsimonious set of predictors for the final model. The assumption of linearity for continuous predictors in the logit was assessed using the Box-Tidwell test. No significant deviations from linearity were found (P > 0.05 for all variables), supporting their inclusion as linear terms in the logistic regression model. Potential influential outliers were examined by calculating Cook’s distance for all observations in the final regression model. No data points with a Cook’s distance greater than 1.0 were identified, suggesting the absence of overly influential cases. A Nomogram model was constructed using RStudio 3.4 software. Statistical significance was set at P<0.05.

### 2.5 Model performance and validation

The model’s performance was evaluated for both discrimination and calibration. Discrimination, the ability of the model to distinguish between patients who did and did not develop hypocalcemia, was assessed using the area under the receiver operating characteristic curve (AUC). To correct for potential overfitting and provide a more realistic estimate of performance, an internal validation was performed using a bootstrapping technique with 1000 resamples. Calibration, which measures the agreement between predicted probabilities and observed outcomes, was assessed graphically with a calibration plot and statistically with the Hosmer-Lemeshow goodness-of-fit test. A non-significant P-value (P > 0.05) in the Hosmer-Lemeshow test indicates good calibration. The overall performance of the model was quantified using the Brier score, which measures the mean squared difference between predicted probabilities and actual outcomes (a lower score indicates better performance). The clinical utility of the nomogram was evaluated using decision curve analysis (DCA), which assesses the net benefit of using the model to make clinical decisions across a range of risk thresholds.

## 3 Results

### 3.1 General characteristics and incidence of postoperative hypocalcemia in DTC patients

Among 315 DTC patients, there were 104 males and 211 females, with an age range of 21–76 years (mean ± SD: 56.89 ± 9.75 years). A total of 237 patients were married, and 78 were unmarried/divorced/widowed. The body mass index (BMI) ranged from 17.63 to 25.12 kg/m², with a mean of 23.02 ± 1.89 kg/m². Postoperative hypocalcemia occurred in 69 patients, and severe hypocalcemia in 32 patients, resulting in an overall incidence of 32.06% (n=101).

### 3.2 Univariate analysis of risk factors for postoperative hypocalcemia in DTC patients

The proportions of diabetes mellitus, central lymph node dissection, and capsular invasion were significantly higher in the occurrence group than in the non-occurrence group (P<0.05). Similarly, operation time, and preoperative serum levels of ALB and Lp-PLA2 were significantly higher in the occurrence group (P<0.05). Conversely, preoperative serum Nesfatin-1 levels were significantly lower in the occurrence group (P<0.05). See **Table 1**.

**Table 1:** Univariate Analysis of Risk Factors for Postoperative Hypocalcemia in DTC Patients.

Clinical Characteristics	Occurrence Group (n=101)	Non-Occurrence Group (n=214)	χ²/t	P
Male/Female (n)	32/69	72/142	0.181	0.670
Age [n(%)]			0.201	0.904
<45 years	27 (26.73)	53 (24.77)		
45-60 years	47 (46.53)	104 (48.60)		
>60 years	27 (26.73)	57 (26.64)		
Marital Status [n(%)]			0.120	0.728
Married	75 (74.26)	162 (75.70)		
Unmarried/Divorced/Widowed	26 (25.74)	52 (24.30)		
BMI [n(%)]			0.918	0.632
<18.5 kg/m²	14 (13.86)	34 (15.89)		
18.5-24.9 kg/m²	61 (60.40)	137 (64.02)		
>24.9 kg/m²	26 (25.74)	43 (20.09)		
Hypertension [n(%)]	25 (24.75)	48 (22.43)	0.191	0.662
Diabetes [n(%)]	26 (25.74)	33 (15.42)	4.621	0.031
Hyperlipidemia [n(%)]	45 (44.55)	89 (41.59)	0.211	0.646
Tumor Diameter [n(%)]			0.412	0.521
<2 cm	20 (19.80)	35 (16.36)		
≥2 cm	81 (80.20)	179 (83.64)		
Lesion Scope [n(%)]			2.987	0.084
Unilateral	29 (28.71)	83 (38.78)		
Bilateral	72 (71.29)	131 (61.22)		
Surgical Approach [n(%)]			0.112	0.946
Endoscopic Surgery	35 (34.65)	76 (35.51)		
Da Vinci Robot Surgery	49 (48.51)	99 (46.26)		
Traditional Surgery	17 (16.83)	39 (18.22)		
Lateral Cervical Lymph Node Dissection [n(%)]	46 (45.54)	94 (43.93)	0.571	0.486
Central Lymph Node Dissection [n(%)]	56 (55.45)	90 (42.06)	4.382	0.036
Capsular Invasion [n(%)]	29 (28.71)	30 (14.02)	8.583	0.003
Operation Time (min)	83.12±10.28	76.95±8.49	5.521*	<0.001
ALB (g/L)	41.79±4.62	35.32±3.25	13.782*	<0.001
Nesfatin-1 (ng/ml)	5.10±1.21	7.42±1.83	10.614*	<0.001
Lp-PLA2 (ng/ml)	22.08±4.15	17.05±3.61	10.623*	<0.001

ALB. Albumin; Nesfatin-1. New satiety molecule protein-1; Lp-PLA2. Lipoprotein-associated phospholipase A2; * indicates t-value.

### 3.3 Multivariate analysis of independent risk factors for postoperative hypocalcemia in DTC patients

Using multivariate logistic regression analysis with postoperative hypocalcemia as the dependent variable (non-occurrence=0, occurrence=1) and variables selected via the backward stepwise procedure, diabetes, central lymph node dissection, capsular invasion, operation time, Lp-PLA2, and ALB were identified as independent risk factors, while Nesfatin-1 was a protective factor (P<0.05). See **Table 2**.

**Table 2:** Multivariate Analysis of Risk Factors for Postoperative Hypocalcemia in DTC Patients.

Variable	β	S.E.	OR	95% CI	Wald χ²	P
Constant	-2.135	0.428	–	–	24.869	<0.001
Diabetes (yes vs no)	0.742	0.251	2.1	1.4–3.3	8.847	0.003
Central Lymph Node Dissection (yes vs no)	1.224	0.305	3.4	2.1–5.6	16.052	<0.001
Capsular Invasion (yes vs no)	1.029	0.321	2.8	1.7–4.7	10.337	0.001
Operation Time (per 10 min)	0.064	0.021	1.9	1.3–3.0	9.357	0.002
ALB (per 1 g/L)	0.531	0.162	1.7	1.2–2.5	10.876	0.001
Lp-PLA2 (per 1 ng/ml)	0.833	0.287	2.3	1.5–3.7	8.465	0.004
Nesfatin-1 (per 1 ng/ml)	-0.511	0.193	0.6	0.4–0.9	7.032	0.008

ALB. Albumin; Nesfatin-1. New satiety molecule protein-1; Lp-PLA2. Lipoprotein-associated phospholipase A2. The OR for Operation Time is presented for every 10-minute increment.

### 3.4 Construction and validation of a nomogram prediction model for postoperative hypocalcemia in DTC patients

**Figure 1:**
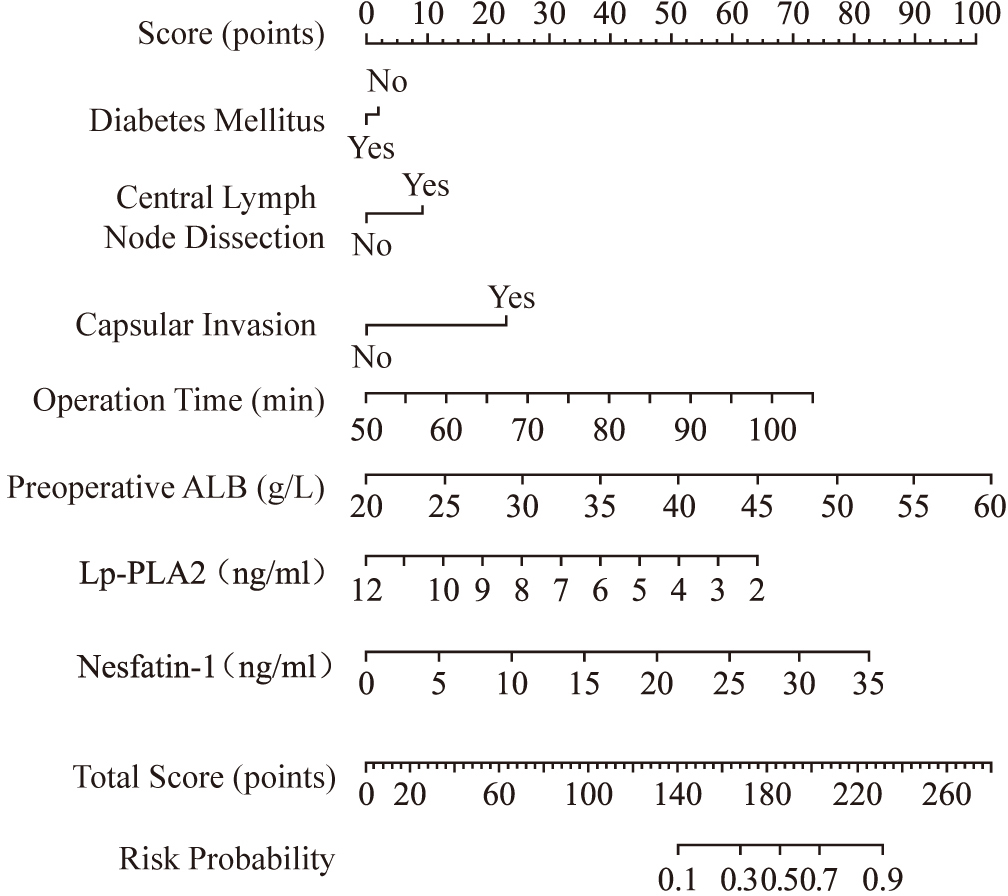
Nomogram risk prediction model diagram.

**Figure 2:**
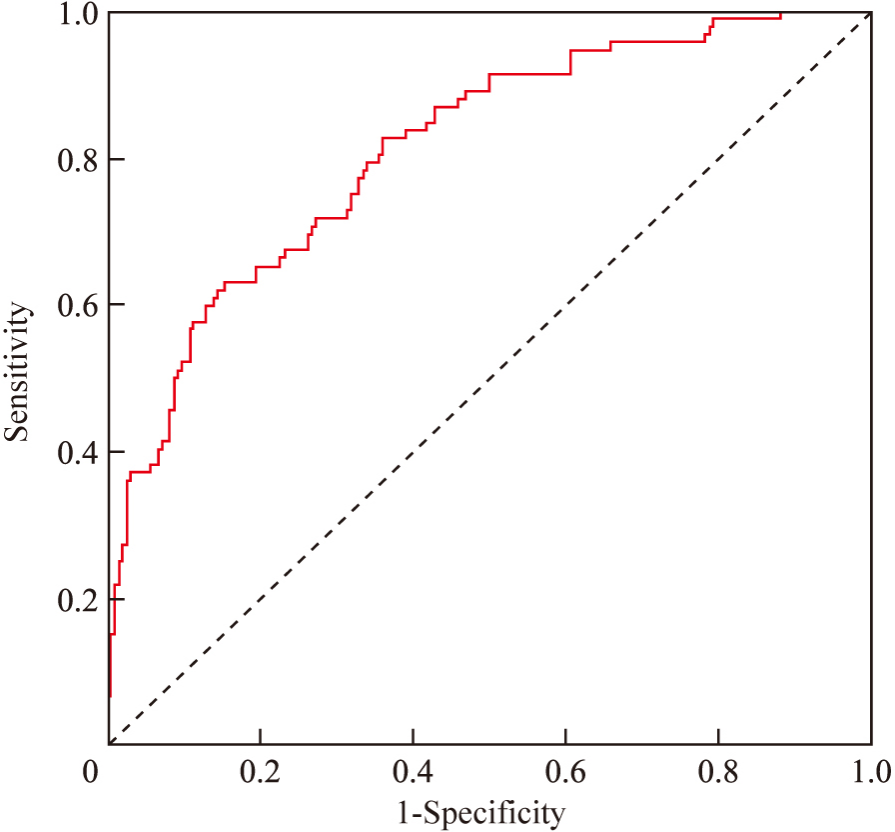
ROC curve analysis of the nomogram risk prediction model.

**Figure 3:**
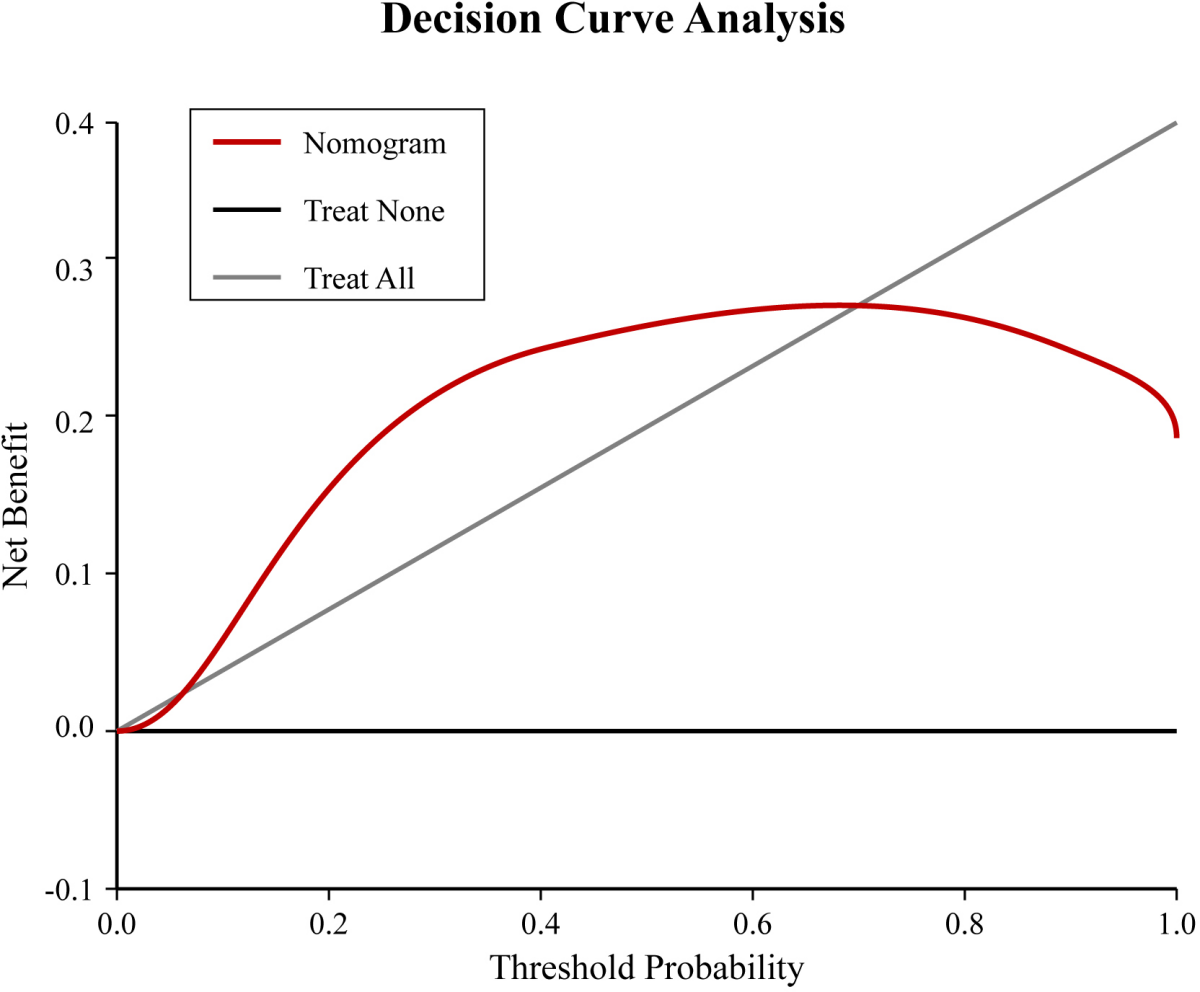
Calibration plot for the nomogram. The x-axis represents the nomogram-predicted probability of postoperative hypocalcemia, and the y-axis represents the observed actual probability. The diagonal dashed line represents a perfect prediction. The solid line represents the performance of the nomogram, with closer proximity to the dashed line indicating better calibration. The shaded area represents the 95% confidence interval for the calibration curve.

**Figure 4:**
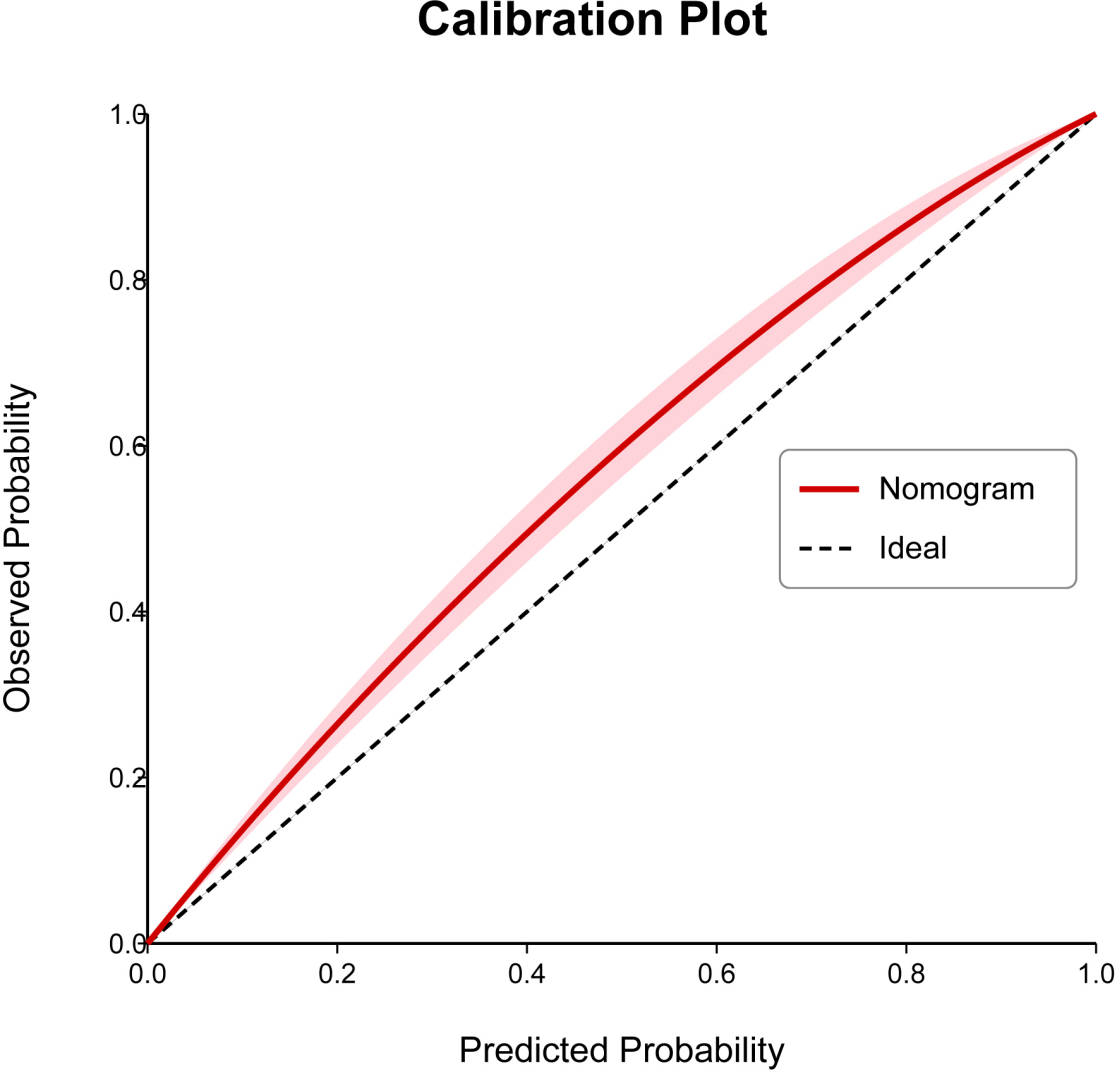
Decision curve analysis (DCA) for the nomogram. The x-axis represents the threshold probability, and the y-axis represents the net benefit. The red line represents the nomogram. The gray line represents the assumption that all patients will develop hypocalcemia (treat all). The black line represents the assumption that no patient will develop hypocalcemia (treat none). The nomogram shows a positive net benefit over a wide range of threshold probabilities compared to the treat-all or treat-none strategies.

A nomogram risk prediction model was constructed based on the results of the multivariate logistic regression (**Figure 1**). A receiver operating characteristic (ROC) curve was generated to evaluate its discriminative ability (**Figure 2**). The area under the ROC curve (AUC) of the Nomogram model was 0.850 (95%CI 0.825–0.930), indicating good discriminative ability for predicting postoperative hypocalcemia in DTC patients. After internal validation using 1000 bootstrap resamples, the bias-corrected AUC was 0.845, suggesting minimal overfitting. The nomogram also showed good calibration, as confirmed by the Hosmer-Lemeshow test (χ²=9.54, P=0.30), indicating no significant difference between predicted and observed frequencies. The calibration plot further visualized this agreement, with the calibration curve closely approximating the ideal 45-degree diagonal line (**Figure 3**). The Brier score for the model was 0.162, indicating a high level of overall accuracy. Decision curve analysis demonstrated that using the nomogram to predict postoperative hypocalcemia offered a greater net benefit than treating all or no patients across a wide range of threshold probabilities (from approximately 10% to 68%), confirming its clinical utility (**Figure 4**).

## 4 Discussion

Hypocalcemia is a major manifestation of hypoparathyroidism after thyroid cancer surgery, which can cause perioral numbness, tetany, and even tracheal spasm leading to asphyxiation, posing adverse effects on patients’ physical and mental health (9, 10). This study showed that the incidence of postoperative hypocalcemia in 315 patients with differentiated thyroid cancer was 32.06%, lower than the 42.13% reported by Tang et al. (11) but higher than the 27.65% reported by Chen et al. (12). This discrepancy may be attributed to differences in the primary diseases of the study population and the criteria for defining hypocalcemia.

In recent years, studies have confirmed that factors such as inadvertent parathyroid gland resection or devascularization are the primary risk factors for hypocalcemia after thyroid-related surgeries (13, 14). In addition to these factors, this study identified that bilateral lesions, central lymph node dissection, capsular invasion, operation time, diabetes, and preoperative serum levels of ALB, Lp-PLA2, and Nesfatin-1 are influencing factors for hypocalcemia after DTC surgery. From the perspective of lesion scope, the wider resection required for bilateral lesions increases the likelihood of parathyroid injury, thereby elevating the risk of postoperative hypocalcemia (15). Previous research has indicated that central lymph node dissection can disrupt the blood supply to the parathyroid glands, leading to abnormal parathyroid hormone secretion and hypocalcemia (16), which aligns with our findings. The parathyroid glands are located between the true and false capsules of the thyroid gland. During thyroid resection, delicate operations must be performed within the narrow space between these capsules. When the capsule is invaded by tumor cells, surgical difficulty significantly increases, which greatly increases the likelihood of parathyroid injury and elevates the risk of postoperative hypocalcemia (17). To address this, ultra-fine capsule dissection techniques have been developed to perform precise operations within the narrow thyroid capsule, preserving parathyroid glands and their blood supply as much as possible. This approach helps reduce parathyroid injury caused by capsule invasion and lowers the incidence of hypocalcemia. However, it also has limitations, such as restricted field of view, natural hand tremors of surgeons, and visual impairments, which can affect the precision of operations. Previous studies have confirmed a correlation between operation time and tissue damage (18). Our study showed that prolonged operation time increases the risk of postoperative hypocalcemia, as it directly affects the duration of tissue exposure and the degree of tissue trauma, increasing the risk of parathyroid injury and thus becoming a risk factor for hypocalcemia.

ALB, the most abundant protein in plasma, is an important carrier protein for blood calcium ions (19). In this study, higher preoperative ALB levels were identified as a risk factor for postoperative hypocalcemia. This seemingly counterintuitive finding may be explained by postoperative physiological changes. Following major surgery, an acute inflammatory response often leads to a significant drop in serum albumin levels due to capillary leakage and decreased synthesis (20). In patients with high preoperative albumin, this postoperative drop may be more pronounced in absolute terms, causing a larger corresponding decrease in total serum calcium, as approximately 40-50% of calcium is albumin-bound. This larger fluctuation could increase the likelihood of transiently reaching the hypocalcemic threshold, especially when parathyroid function is temporarily compromised. Furthermore, higher preoperative albumin could reflect a better nutritional state and more active bone metabolism, potentially predisposing patients to a more severe ‘hungry bone syndrome’ post-surgery (21, 22). Surveys have shown that compared with healthy individuals, patients with type 2 diabetes have a twofold increased risk of thyroid dysfunction (23). Glucose metabolism disorders can affect the endocrine system, leading to thyroid hormone synthesis and storage disorders. Additionally, diabetes-related microvascular complications may impair parathyroid blood supply, rendering the glands more susceptible to ischemic injury during surgery and thus becoming a risk factor for hypocalcemia after DTC surgery (24, 25).

Lp-PLA2 is a leukocyte-derived enzyme that is released in large quantities during inflammatory responses. It exerts pro-inflammatory effects by hydrolyzing low-density lipoproteins and is involved not only in atherosclerosis but also in the dysfunction of organs such as the thyroid and kidneys (26). Nesfatin-1 is a satiety molecule that improves insulin resistance (27). Insulin resistance is the pathological basis of type 2 diabetes, and patients with type 2 diabetes often have dysfunction of the hypothalamic-pituitary-thyroid axis. Additionally, the thyroid gland is susceptible to high glucose toxicity, increasing the risk of thyroid pathology indirectly linked to the endocrine milieu that may affect calcium homeostasis (28, 29). Accurately assessing the influencing factors of postoperative hypocalcemia and implementing feasible prevention and control measures to reduce its adverse effects are important tasks in clinical treatment (30, 31).

In conclusion, this study identified that central lymph node dissection, capsular invasion, operation time, diabetes, and preoperative serum levels of ALB, Lp-PLA2, and Nesfatin-1 are independent predictors for hypocalcemia after DTC surgery. The Nomogram risk prediction model demonstrates good discrimination, calibration, and clinical utility in predicting postoperative hypocalcemia. However, this study has several limitations. First, as a single-center retrospective study, selection bias is inherent, and the conclusions may have certain biases. Although internal validation using bootstrapping showed good model stability, the lack of external validation in a separate, multicenter cohort remains a significant limitation, and the model’s generalizability is currently unknown. Second, we did not perform a sensitivity analysis, for instance, by excluding patients with mild hypocalcemia, to assess the robustness of our findings. Third, we analyzed several biochemical markers without formal correction for multiple comparisons, which increases the risk of Type I error. Therefore, the associations of Lp-PLA2 and Nesfatin-1 with hypocalcemia should be interpreted with caution and require validation in independent studies. Finally, despite including a wide range of variables, there may be residual confounding from unmeasured factors, such as vitamin D levels or the specific surgical techniques of individual surgeons. Future studies should aim to address these limitations by conducting large-scale, prospective, multicenter validations.

## Data Availability

The original contributions presented in the study are included in the article/**Supplementary Material**. Further inquiries can be directed to the corresponding author.

## Supplementary material

Supplementary Figure 1 Flowchart of the study. The diagram illustrates the patient selection process, data collection, statistical analysis workflow, and the development and validation steps for the nomogram.

## References

[1] Aqtashi B Ahmad N Frotzler A Bähler S Linder T Müller W. Risk factors for hypocalcaemia after completion hemithyroidectomy in thyroid cancer. Swiss Med weekly. (2017) 147:w14513. doi: 10.4414/smw.2017.14513, PMID: 29120026

[2] Qin X Luo J Ma J Cao X Zhao J Jiang J. Prospective cohort study of parathyroid function and quality of life after total thyroidectomy for thyroid cancer: robotic surgery vs. open surgery. Int J Surg (London England). (2023) 109:3974–82. doi: 10.1097/JS9.0000000000000725, PMID: 37755372 PMC10720820

[3] Shoback DM Bilezikian JP Costa AG Dempster D Dralle H Khan AA. Presentation of hypoparathyroidism: etiologies and clinical features. J Clin Endocrinol Metab. (2016) 101:2300–12. doi: 10.1210/jc.2015-3909, PMID: 26943721

[4] Sitges-Serra A Gallego-Otaegui L Suárez S Lorente-Poch L Munné A Sancho J. Inadvertent parathyroidectomy during total thyroidectomy and central neck dissection for papillary thyroid carcinoma. Surgery. (2017) 161:712. doi: 10.1016/j.surg.2016.08.021, PMID: 27743717

[5] Dolidze D Shabunin A Vardanyan A Melnik K Covantsev S. Prophylaxis of postoperative hypoparathyroidism in thyroid surgery. Folia Med (Plovdiv). (2023) 65:207–14. doi: 10.3897/folmed.65.e75427, PMID: 37144304

[6] Huang DN Li ZD Liu HW. Analysis of influencing factors of hypocalcemia after initial total thyroidectomy for thyroid cancer. Clin J Med Off. (2022) 50:802–5.

[7] Tang J Jiang S Gao L Xi X Zhao R Lai X. Construction and validation of a nomogram based on the log odds of positive lymph nodes to predict the prognosis of medullary thyroid carcinoma after surgery. Ann Surg Oncol. (2021) 28:4360–70. doi: 10.1245/s10434-020-09567-3, PMID: 33469797

[8] Lukinović J Bilić M. Overview of thyroid surgery complications. Acta Clin Croat. (2020) 59:81–6. doi: 10.20471/acc.2020.59.s1.10, PMID: 34219888 PMC8212606

[9] Singh J Bhardwaj B. Effect of microdissection of inferior thyroid artery on post-operative hypocalcemia in total thyroidectomy. Indian J Otolaryngol Head Neck Surg. (2023) 75:1461–8. doi: 10.1007/s12070-023-03576-w, PMID: 37636650 PMC10447685

[10] He L. Analysis of related factors of postoperative transient hypoparathyroidism in thyroid cancer patients. Chin Remedies Clin. (2022) 22:372–5.

[11] Jiang WJ Yan PJ Zhao CL Si MB Tian W Zhang YJ. Comparison of total endoscopic with conventional open thyroidectomy for treatment of papillary thyroid cancer: a systematic review and meta-analysis. Surg Endosc. (2020) 34(5):1891–903. doi: 10.1007/s00464-019-07283-y, PMID: 32144555

[12] Chen SY Pan B Lei X. Risk factors for early hypocalcemia after surgery for secondary hyperparathyroidism in dialysis patients. J Nephrol Dial Transplant. (2023) 32:214–9.

[13] Unlu MT Kostek M Caliskan O Aygun N Uludag M. Does the risk of hypocalcemia increase in complementary thyroidectomy performed in papillary thyroid cancer? Sisli Etfal Hastan Tip Bul. (2022) 56(4):482–8. doi: 10.14744/SEMB.2022.91073, PMID: 36660383 PMC9833338

[14] Xu GD Ling YW Zhu J. A nomogram prediction model for delayed hypoparathyroidism after thyroid cancer surgery. Chin J Gen Surg Basic Clin. (2022) 29:24–31.

[15] Orloff LA Wiseman SM Bernet V Fahey TJ 3rd Shaha AR Shindo ML. American thyroid association statement on postoperative hypoparathyroidism: diagnosis, prevention, and management in adults. Thyroid: Off J Am Thyroid Assoc. (2018) 28(7):830–41. doi: 10.1089/thy.2017.0309, PMID: 29848235

[16] Yan J. Effects of total thyroidectomy on stress response, recurrent laryngeal nerve, and parathyroid gland injury in patients with bilateral thyroid cancer. Shaanxi Med J. (2022) 51:831–4.

[17] Leboulleux S Bournaud C Chougnet CN Zerdoud S Al Ghuzlan A Catargi B. Thyroidectomy without radioiodine in patients with low-risk thyroid cancer. N Engl J Med. (2022) 366(10):923–32. doi: 10.1056/NEJMoa2111953, PMID: 35263518

[18] Van Slycke S Van den Heede K Brusselaers N Vermeersch H. Feasibility of autofluorescence for parathyroid glands during thyroid surgery and the risk of hypocalcemia: First results in Belgium and review of the literature. Surg Innov. (2021) 28(4):409–18. doi: 10.1177/1553350620980263, PMID: 33372584

[19] Patel D Haag S Patel J Ytreberg F Bernards M. Paired simulations and experimental investigations into the calcium-dependent conformation of albumin. J Chem Inf modeling. (2022). doi: 10.1021/acs.jcim.1c01104, PMID: 35194993 PMC9007495

[20] He Q Zhang Q Qian J Yu S Liao L Li Q. Laparoscopic hepatectomy reduces the incidence of postoperative hypoalbuminemia: A propensity score matching analysis with open hepatectomy. Clin Surgery. (2021) 6:3376. doi: 10.47829/COS.2021.61001

[21] Guo YY Zhou P Li XL. Establishment and validation of postoperative risk scoring model for severe hypocalcemia in patients with secondary hyperparathyroidism after surgery. Chin J Gen Surgery. (2022) 31:1414–21.

[22] Zhang HB Zhao HH Wang SX. Perioperative observation and postoperative risk factors of severe hypocalcemia after parathyroidectomy: a report of 303 cases. J Shandong Univ (Health Sciences). (2020) 58:14–20.

[23] Han C He X Xia X Li Y Shi X Shan Z. Subclinical hypothyroidism and type 2 diabetes: A systematic review and meta-analysis. PloS One. (2015) 10(8):e0135233. doi: 10.1371/journal.pone.0135233, PMID: 26270348 PMC4535849

[24] Roa Dueñas OH Van der Burgh AC Ittermann T Ligthart S Ikram MA Peeters R. Thyroid function and the risk of prediabetes and type 2 diabetes. J Clin Endocrinol Metab. (2022) 107(6):1789–98. doi: 10.1210/clinem/dgac006, PMID: 35137143 PMC9315162

[25] Passali M Josefsen K Frederiksen JL Antvorskov JC. Current evidence on the efficacy of gluten-free diets in multiple sclerosis, psoriasis, type 1 diabetes and autoimmune thyroid diseases. Nutrients. (2020) 12(8):2316–26. doi: 10.3390/nu12082316, PMID: 32752175 PMC7468712

[26] Fras Z Tršan J Banach M. On the present and future role of Lp-PLA2 in atherosclerosis-related cardiovascular risk prediction and management. Arch Med Science: AMS. (2020) 17:954–64. doi: 10.5114/aoms.2020.98195, PMID: 34336025 PMC8314407

[27] Mohapatra S. Nesfatin-1: a novel satiety factor and its molecular cloning applications. Innov Mol Biotechnol. (2023).

[28] Bos M Smit R Trompet S Van Heemst D Noordam R. Thyroid signaling, insulin resistance, and 2 diabetes mellitus: A mendelian randomization study. J Clin Endocrinol Metab. (2017) 102:1960. doi: 10.1210/jc.2016-2816, PMID: 28323940

[29] Yang W Jin C Wang H Lai Y Li J Shan Z. Subclinical hypothyroidism increases insulin resistance in normoglycemic people. Front endocrinol. (2023) 14. doi: 10.3389/fendo.2023.1106968, PMID: 37484968 PMC10358968

[30] Antakia R Edafe O Uttley L Balasubramanian S. Effectiveness of preventative and other surgical measures on hypocalcemia following bilateral thyroid surgery: a systematic review and meta-analysis. Thyroid: Off J Am Thyroid Assoc. (2015) 25 1:95–106., PMID: 25203484 10.1089/thy.2014.0101

[31] Docimo G Ruggiero R Casalino G Del Genio G Docimo L Tolone S. Risk factors for postoperative hypocalcemia. Updates Surgery. (2017) 69:255–60. doi: 10.1007/s13304-017-0452-x, PMID: 28444542

