# Supplementary figures and images for: Development and validation of a nomogram for predicting postoperative hypocalcemia in patients undergoing surgery for differentiated thyroid cancer
Source: Front Endocrinol (Lausanne). 2025 Oct 6;16:1628453. doi: 10.3389/fendo.2025.1628453 (PMC12535898; doi:10.3389/fendo.2025.1628453)

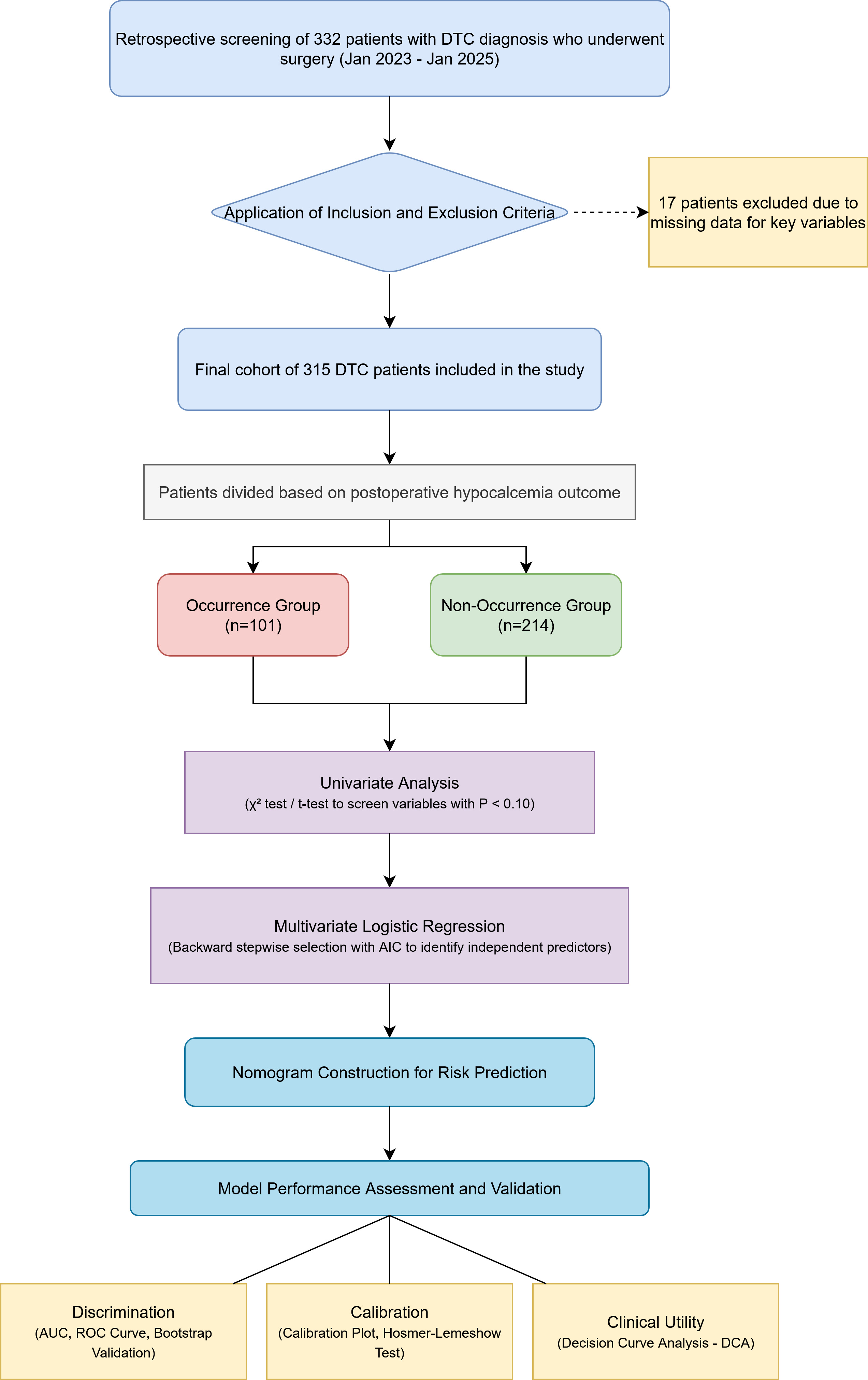

Supplement: Supplementary Figure 1 — Flowchart of the study. The diagram illustrates the patient selection process, data collection, statistical analysis workflow, and the development and validation steps for the nomogram. [file Image1.jpg]
